# Distribution characteristics of Purkinje fibres in the canine left ventricle

**DOI:** 10.1111/jcmm.70117

**Published:** 2024-09-25

**Authors:** Yunhao Li, Daoyang Zhang, Yunfan Meng, Jie Zhang, Qi Zhang, Ping Zhang, Yujie Zhang, Mingyu Sun, Zulu Wang, Ming Liang

**Affiliations:** ^1^ Department of Cardiology The General Hospital of Northern Theater Command Shenyang Liaoning China; ^2^ Graduate School of China Medical University China Medical University Shenyang Liaoning China; ^3^ Graduate School of Dalian Medical University Dalian Medical University Dalian Liaoning China; ^4^ National Key Laboratory of Frigid Zone Cardiovascular Diseases Shenyang Liaoning China

**Keywords:** anatomy, iodine staining, morphology, purkinje fibres

## Abstract

Purkinje‐related ventricular arrhythmias have been increasingly reported, and with the development of catheter ablation techniques, intervention for Purkinje‐related arrhythmias has been shown to be effective. The characteristics of Purkinje fibres orientation in the 12 canine left ventricles were observed at a gross level by staining the endocardium with Lugol's solution. Purkinje fibres were observed microscopically by HE, Masson's, PAS glycogen, and Cx40 immunohistochemical staining. Staining was successful, and the transverse orientation characteristics of Purkinje fibres were observed by Lugol's staining, and the longitudinal distribution was observed microscopically. The distribution of Purkinje fibres in the canine left ventricle is ‘graded’, ‘layered’, and ‘networked’, which can guide catheter ablation of Purkinje‐related ventricular arrhythmia.

## 1 INTRODUCTION

The cardiac conduction system comprises specialized cardiomyocytes that regulate the coordinated activity of the heart. The Purkinje fibre network, located beneath the endocardium, is the terminal part of the cardiac conduction system and was first discovered and named by Johannes Purkinje of Czechoslovakia in 1825.^1^ Originating from the ventricular base, Purkinje fibres form a series of bundles in the subendocardial layer that gradually converge and cross to extend towards the apical region of the heart. These bundles intertwine to form a network within the subendocardium extending perpendicularly to the endocardial surface between the myocardium and the working cells. Studies have suggested that the development and persistence of certain ventricular tachycardias may be related to structural or functional abnormalities within the Purkinje fibre network.^2^, ^3^ Purkinje fibres carry faster conduction and shorter action potential durations compared to ventricular myocytes and play a fundamental role in enabling the ventricular myocardium to contract in a coordinated manner as a functional syncytium. Although multiple Purkinje potentials are often recorded in vivo studies, their anatomical characteristics remain unknown, hampering the study and research of their electrophysiological mechanisms. The aim of this study was to provide an overview of the anatomical features of Purkinje fibres in the canine left ventricle by gross and microscopic observations, with the aim of guiding experimental and catheter ablation interventions for Purkinje‐associated ventricular arrhythmias.

## 2 MATERIALS AND METHODS

### 2.1 Materials

Twelve beagles weighing 21.5 ± 3 kg were euthanized under midazolam anaesthesia (0.5 mg/kg) and 150 mL air was injected into the femoral vein. Immediately after the execution, a lateral incision was made along the sternum to obtain 12 fresh beagle hearts with the lungs still attached to ensure the integrity of the atria and ventricles. Within 30 min of canine death, the experimental hearts were prepared and preconditioned. The major blood vessels were dissected approximately 5 cm before entering the ventricles. The left atrium was cut through the pulmonary vein, and an incision was made in the anterolateral wall of the left ventricle between the anterior and posterior groups of papillary muscles, extending to the apex of the heart. The aorta was then incised between the left and posterior aortic valves, fully exposing the left ventricle. The endocardium was carefully cleaned of any remaining blood clots, and the prepared hearts were immersed in iced lactated Ringer's sodium solution to maintain their activity. The canines used in the study were provided by Changzhou Beile Laboratory Animal Breeding Co Ltd. (licence no: SCXK (Su) 2018–0007). The experimental protocol was approved by the Institutional Animal Care and Use Committee. All procedures involving experimental animals during the study were conducted in accordance with the Code of Ethics of the World Medical Association (Declaration of Helsinki), and the study was approved under ethical approval number SH2022‐06008.

### 2.2 Staining

Lugol's solution was prepared in advance by dissolving 4 g iodine and 4 g potassium iodide in 100 mL deionized water at room temperature. The solution was stored away from light. Lugol's solution was evenly sprayed onto the prepared endocardium and allowed to soak for approximately 2 min to facilitate staining of the Purkinje fibre network.

After staining, the sections were fixed in 5% formaldehyde solution. They were then embedded in paraffin and cut perpendicular to the endocardium to obtain histological sections with a thickness of 5 μm. These sections were stained using HE, Masson's, PAS, and Cx40 immunohistochemical techniques.

### 2.3 Statistical methods

SPSS 26.0 software was utilized for statistical analysis. Continuous variables that exhibited a normal distribution are presented as $\bar{x}\pm s$.

## 3 RESULTS

Lugol's fluid staining of the canine endocardium provides a clear visualization of the conduction system and its branches. The orientation of Purkinje fibres in the left ventricle of the canine heart appears to be ‘graded’, ‘layered’ and ‘networked’.

### 3.1 Graded

#### 3.1.1 The trunk of the left bundle branch

**FIGURE 1:**
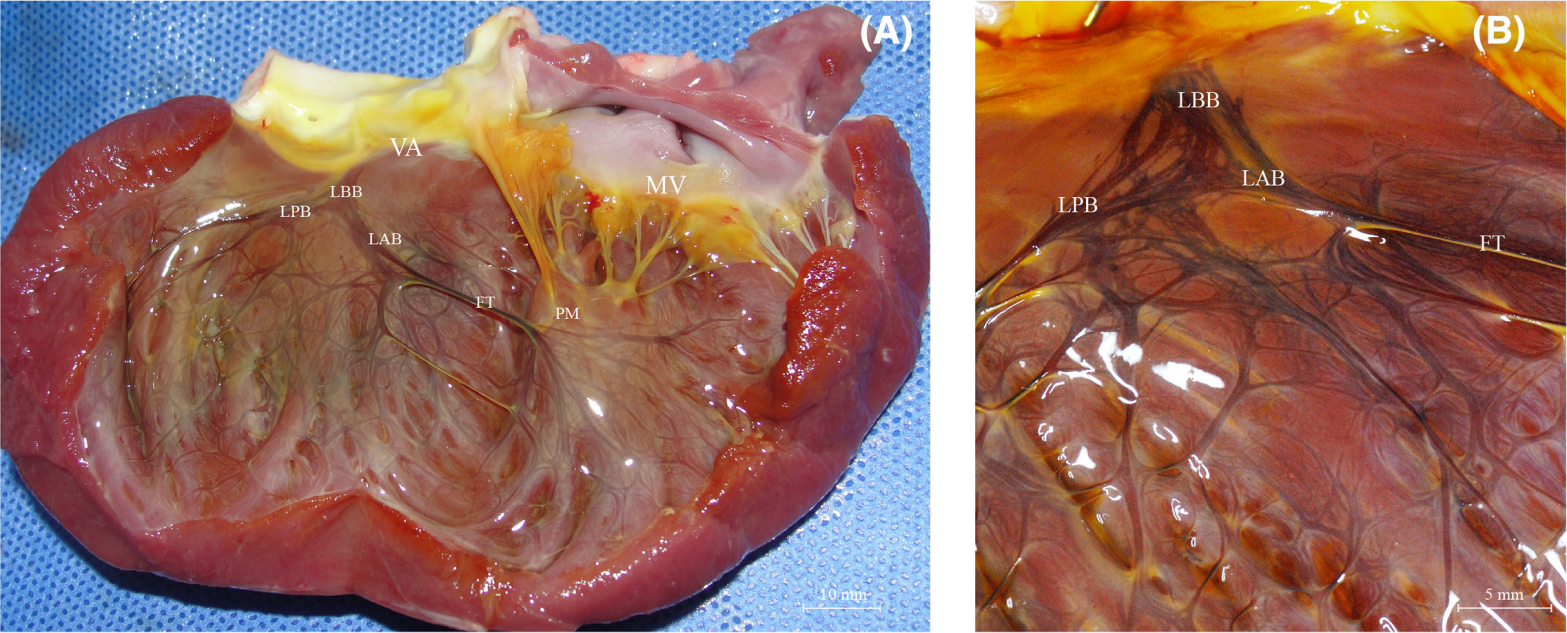
(A) Canine left ventricular Purkinje fibres follow a graded course in the order of the trunk of the left bundle branch, the LAB and LPB, the network of secondary Purkinje fibres and the endings of the conduction system; (B) The origin of the trunk of left bundle branch from under the valve annulus. LBB, left bundle branch; LAB, left anterior branch; LPB, left posterior branch; FT, false tendon; PM, papillary muscles; MV, mitral valve; VA, valve aortae; CB, connective bundle.

The trunk of the left bundle branch originates between the right and left coronary sinuses below the aortic valve. It then travels along the left ventricular septum in an apical direction for a mean distance of 9.56 ± 2.64 mm. At this point, it gives rise to two secondary branches, the left anterior branch (LAB) and the left posterior branch (LPB), which form a right angle or obtuse angle with the main trunk. Additionally, conduction bundles are observed along the septal surface (Figure 1A). The main trunk of the left bundle branch consists mainly of numerous thin, parallel conduction bundles with fissure‐like gaps between them (Figure 1B).

#### 3.1.2 Left anterior branch and the left posterior branch

LAB originates from the main trunk of the left bundle branch and adheres to the endocardial surface for an average distance of 23.35 ± 3.22 mm. Part of the LAB gradually extends to the false tendons, while the remaining part continues to run along the endocardial surface. The LAB gives rise to subbranches that form an intertwined Purkinje network. This network appears to be denser near the apical region and more sparsely distributed near the valve annulus.

Similarly, the LPB also travels along the endocardium with a trunk length of approximately 23.46 ± 2.37 mm. The LPB runs subparallel to the edge of the aortic valve, gradually extending into the false tendons and the endocardial surface before converging apically by various routes. The origin and termination of the false tendons carrying the LPB show considerable variation among individuals (Figure 1A).

#### 3.1.3 Septal bundles

The spatial surface between the LAB and LPB exhibits small bundles connecting them, forming a ‘fishing net’ distribution pattern (Figure 1A). These bundles are characterized by numerous subcircular pores that are small and irregular in shape. No large branches of similar thickness to the LAB and LPB were observed. However, there are lateral connecting bundles that provide connections between the LAB and LPB. These bundles extend apically to the crista of the longitudinal trabeculae carneae of the ventricular wall and gradually give rise to smaller branches that cross and converge on the surface of the longitudinal meatus. This arrangement forms a network through the transverse connection of false tendons.

### 3.2 Layered

**FIGURE 2:**
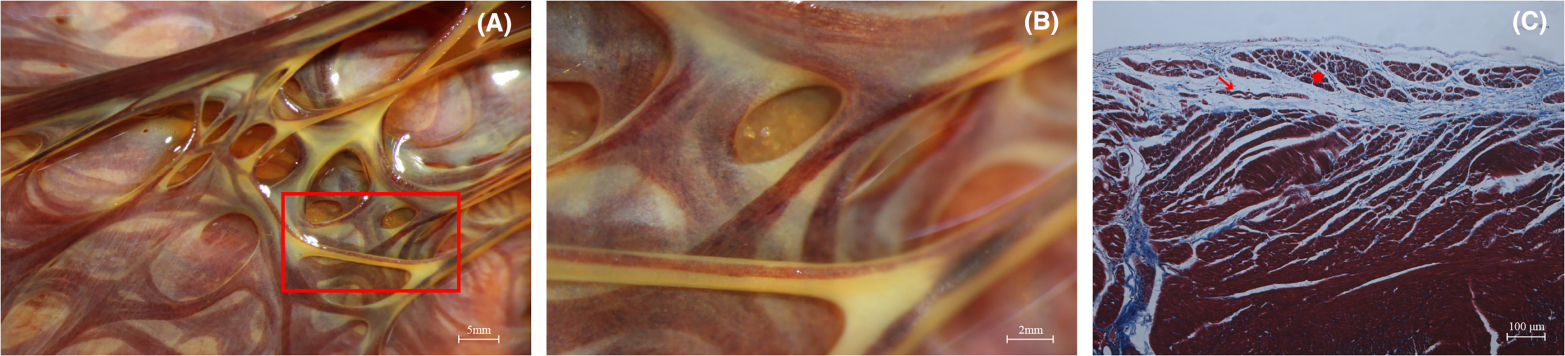
(A) Purkinje fibres are layered and interconnected on the endocardial surface; (B) Local magnification of the red rectangular part of (A); (C) Microscopically, Purkinje fibres are seen to be layered on the endocardial surface. ☆: Purkinje fibres along the short‐axis section; →: Purkinje fibres along the long‐axis section.

Purkinje fibres traversing the endocardial surface show a three‐dimensional distribution in layers perpendicular to the direction of the endocardial surface (Figure 2A,B). Lugol's staining shows that the Purkinje network is distributed at different levels (Figure 2C). Samples were taken from the subendocardial layer of the canine left ventricle near the apex and subjected to Masson's staining, showing that Purkinje fibres are located between the subendothelium and the myocardium, predominantly within loose connective tissue. Within this layer, Purkinje fibres show distinct orientations at different levels (Figure 2C). The bundles are separated by connective tissue and have a striated appearance with sparse longitudinal arrangement. Conduction bundles are distinct from each other, with collagen fibres acting as barriers at different levels. Within the conduction bundles, there is no obvious interconnection between Purkinje fibres (Figure 2C). Masson's stain highlights the blue‐coloured fibrous connective tissue, while the myocardial tissue and conduction system appear deep brownish red. The ventricular endothelium contains collagen fibres and Purkinje fibres are distributed within the loose fibrous tissue of the subendocardium. In the transverse section, the Purkinje fibres are encased by dense fibrous connective tissue and are divided into secondary conduction bundles of varying sizes, which are closely aligned.

### 3.3 Networked

The conduction bundles within the canine left ventricle show interconnectivity and are intricately woven into a network. Several types of connections between conduction bundles are observed: (1) multiple conduction bundles converge to form the same junction; (2) two conduction bundles are connected transversely by another conduction bundle; and (3) a conduction bundle branches and forms connections with other conduction bundles as it extends. Through these primary pathways, Purkinje fibres form an interconnected and interlaced network characterized by closely spaced junctions resembling a ‘fishing net’ arrangement (Figure 2A,B).

### 3.4 Distribution of Purkinje fibres on other structures

#### 3.4.1 Tendons

**FIGURE 3:**
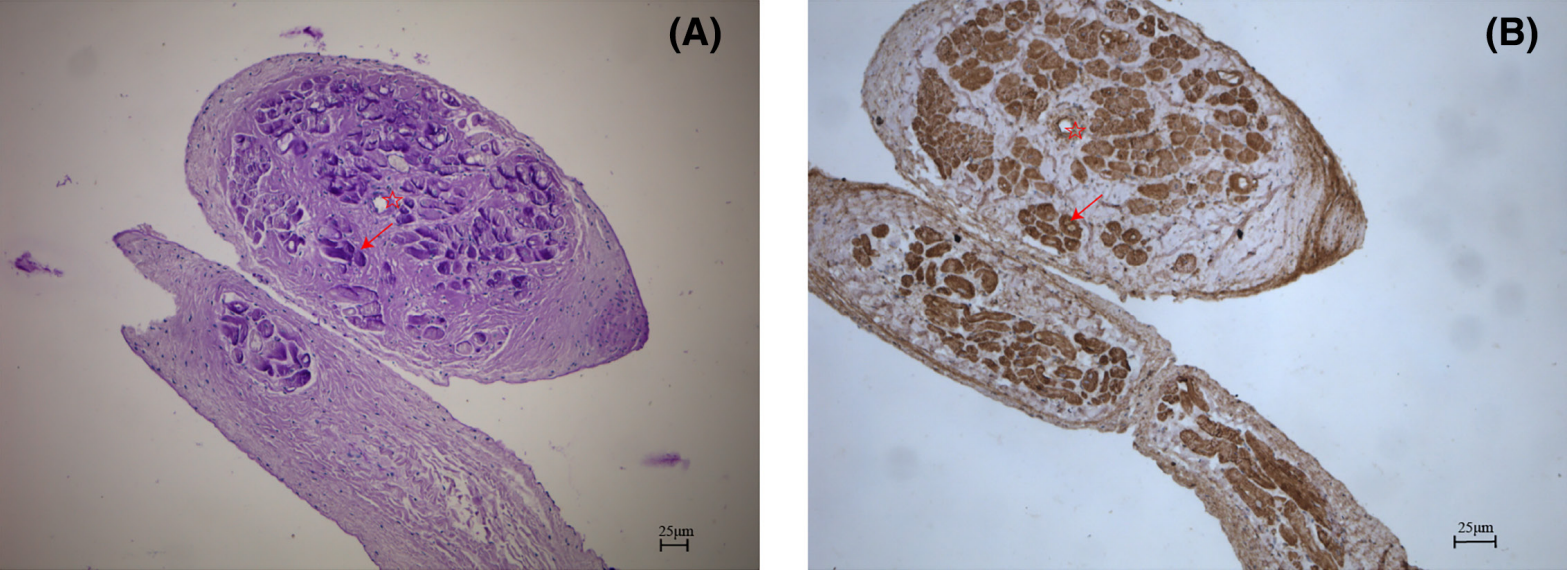
Staining of cross sections of false tendons. (A) PAS glycogen staining, dark blue shows Purkinje fibres running in the false tendons; (B) Cx40 staining, dark brown shows conduction bundles.☆ represents the blood vessels running through the false tendon; the blue‐purple and brown structures indicated by the red arrows are both Purkinje fibres.

**FIGURE 4:**
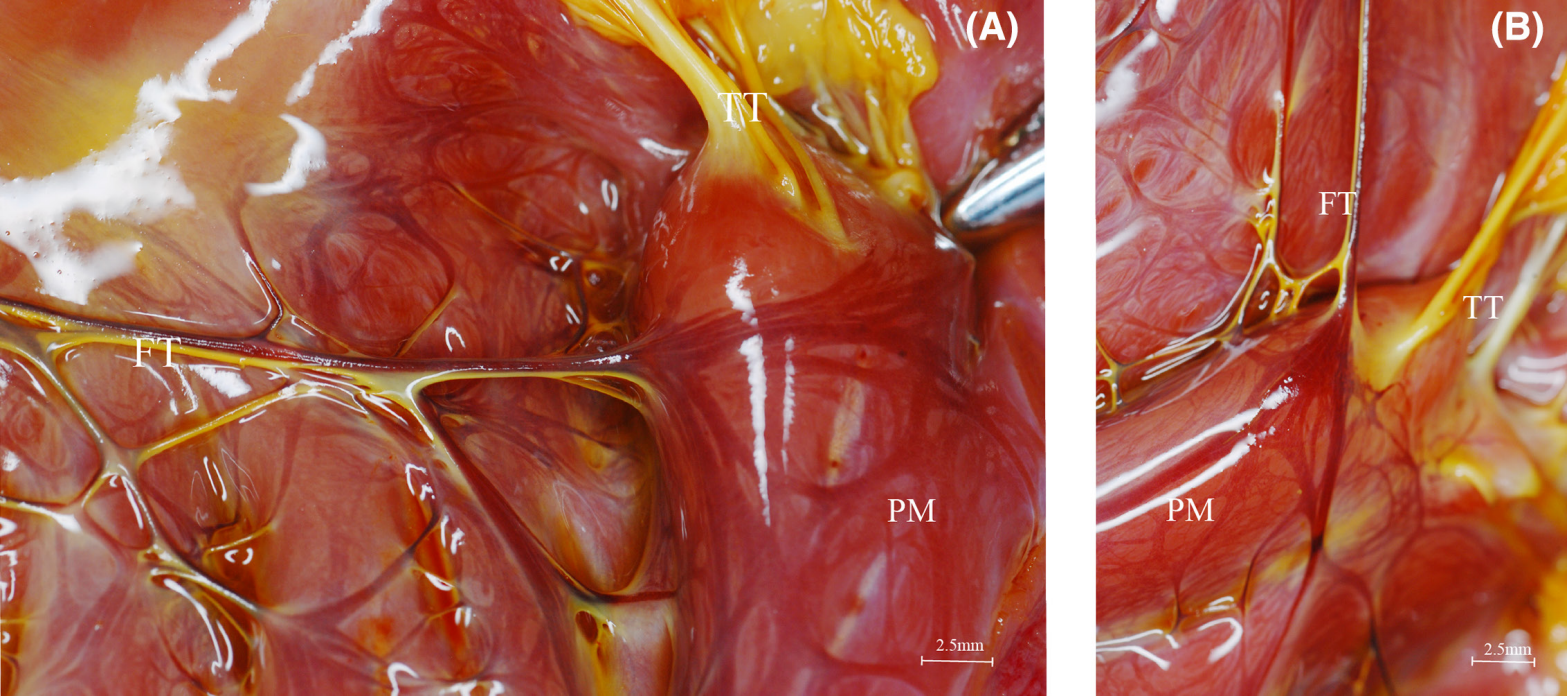
(A) Purkinje fibres at the junction of the false tendon with the endocardium, which fan out after reaching the papillary muscle; (B) Partial magnification of (A), showing the walking Purkinje fibres on the false tendon, which fan out after reaching the papillary muscle, and the absence of the conduction system on the true tendon. FT, false tendons; TT, true tendons; PM, papillary muscle.

The tendons in the left ventricle can be divided into true and false tendons. True tendons connect the papillary muscles to the valve leaflets to prevent excessive stretching of the leaflets when the valve closes. On the other hand, false tendons provide connections between different regions of the ventricle. The orientation of Purkinje fibres within the false chords can be visualized by cross‐sectional staining using PAS and Cx40 immunohistochemistry staining (Figure 3). Purkinje fibres traveling within the false tendons consist of multiple clusters of small conduction bundles that converge or diverge from the false tendons at their ends where they connect to the endocardium (Figure 4A,B). Notably, numerous false tendons less than 0.5 cm in length were observed in the transverse connections between the trabeculae carneae.

#### 3.4.2 Papillary muscles and trabeculae carneae

The papillary muscles serve as conduits for Purkinje fibres. Within the papillary muscles, the anterior and posterior groups are traversed by the LAB and LPB conduction bundles, which are connected by longer false tendons. These false tendons enter the papillary muscle laterally and exhibit a ‘branching’ pattern, giving rise to secondary branches towards the base of the papillary muscle. These branches surround the papillary muscle from the lateral aspect. Additionally, smaller Purkinje branches originate from the circumferential bundles and extend towards the basolateral and apical regions of the heart. Over time, these branches gradually connect with the conduction bundles between the LAB and LPB and the terminal conduction bundles of the posterior branches of the LPB, eventually forming a network (Figure 4A).

The trabeculae carneae originate primarily at the midpoint of the annulus‐apical line and extend longitudinally within the ventricular myocardial septum towards the apical region. These trabeculae carneae vary in size and are separated by longitudinal grooves of varying depth. Lateral false tendons connect the trabeculae carneae, enhancing their structural integrity.

## 4 CONCLUSION AND DISCUSSION

### 4.1 Staining method

The main methods used to visualize the intracardiac conduction system are gross dissection and staining. Gross dissection is particularly suitable for examining larger intracardiac structures such as the sinus node. Conversely, staining techniques are more appropriate for visualizing smaller conduction systems, such as the Purkinje fibres. Various staining techniques are available, including dye perfusion staining, iodine solution staining, and iodine gas staining.^4^, ^5^ Our study opted for Lugol's iodine staining, which takes advantage of the difference in glycogen content between the Purkinje fibres and the endocardial surface to produce different colours.

However, it is important to recognize the limitations of this method. Lugol's iodine stains only the superficial Purkinje fibres, which are located on the endocardium and cannot be stored for long periods. To ensure optimal staining results, canine hearts treated with Lugol's iodine solution should be as fresh as possible, as glycogen degradation over time can affect the staining result. Previous studies by our team^6^ have shown that isolated canine hearts preserved in ice Ringer's solution maintain stable electrical activity for approximately 38–80 min (with an average of 56.5 ± 15.1 min). Therefore, it is imperative that isolated canine hearts are promptly prepared, processed, and stained within this specific time window.

Lugol's solution should be prepared within 1 week of application. Deionized water should be used as the solvent, and potassium iodide and simple iodine should be added sequentially and completely dissolved. It should be noted that Lugol's solution, which has been left unused for a long period of time, will not provide satisfactory staining results.

### 4.2 Distribution characteristics of Purkinje fibres

Purkinje fibres in the canine left ventricle have a graded distribution. As the trunk of the left bundle branch enters the left ventricle, it progressively gives rise to secondary conduction bundles as it extends downward. However, cardiomyocytes close to the trunk of the left bundle branch do not contract initially. This phenomenon may be due to the presence of fibrous connective tissue surrounding these areas, the collagenous components of which have an insulating effect on conduction to some extent. As a result, the superior conduction bundles do not directly stimulate the working myocardial cells, but instead establish a connection through the secondary conduction bundles, which extend to the end of the conduction system.

In the left ventricle, Purkinje fibre follows a graded pattern in which excitation occurs in a fixed sequence: starting from the trunk of the left bundle branch, then the left anterior and posterior branches, the secondary Purkinje fibre network and finally, the end of the conduction system and the myocardial working cells. The research by Romero et al.^7^ indicates that the structural connections between Purkinje fibres and myocardial working cells are relatively complex. The synergistic effect of the Purkinje fibre terminals with certain gap junction proteins can facilitate a more extensive synchronous contraction of myocardial fibres.^8^ This sequential activation allows impulses to propagate rapidly through the Purkinje fibre network, ensuring widespread ventricular excitation in a shorter time. As a result, the ventricular myocardium contracts synchronously, facilitating effective systolic function.

Purkinje fibres show both a layered and a graded distribution. In this study, the ventricular Purkinje fibre network was examined using Masson's staining. The results showed that this network is not a monolayer structure but rather consists of multiple conduction bundles traversing the connective tissue of the subendocardial layers. These bundles are separated into different layers, and there are no distinct bundles connecting the different layers.

The network arrangement of Purkinje fibres of the same grade facilitates rapid impulse propagation throughout the ventricle, ensuring that the working cells of the myocardium receive impulses almost simultaneously. It is important to note that a single conduction bundle does not receive impulses from a single source. Instead, within a conduction bundle, there are various connecting nodes and lateral branches that make connections with other conduction bundles.

### 4.3 Tendons

Tendons can be categorized into false tendons and true tendons. The false tendons are attached to the endocardium, and during development, some of the myocardial working cells gradually transform into conduction system cells. Liang et al.^6^ performed Masson's staining on false tendons and found that the conduction bundles within these tendons were surrounded and separated by fibrous connective tissue. It can be concluded that the false tendons serve as extensions of the conduction bundles on the surface of the endocardium, and the fibrous sheaths surrounding the conduction bundles correspond to the endomysial membrane and the myofascial membrane. These sheaths play a crucial role in organizing Purkinje cells into ‘fibrous’ and ‘branching’ forms, helping to resist mechanical damage and delay conduction.^9^, ^10^, ^11^, ^12^ On the other hand, true tendons are attached to the valve leaflet and limit its movement to some extent. Because of their attachment and function, true tendons experience greater mechanical stretch and have denser collagen fibres than false tendons. Importantly, true tendons do not contain Purkinje fibres.

Purkinje fibres crossing the false tendon have a shorter conduction distance and time. The false tendons, particularly those attached to the left LPB and LAB, act as ‘viaducts’ to accelerate the transmission of impulses to more distant endocardial pathways. These pathways include the free wall of the left ventricle and the anterior and posterior papillary muscles, ultimately ensuring coordinated contraction of the ventricular muscle. Furthermore, the conduction bundles running along the crest of the trabeculae carneae are interconnected by short tendons, forming a polygonal conduction network. This network plays a crucial role in enabling synchronous contraction of most of the ventricles.

### 4.4 Guidance on catheter ablation of cardiac arrhythmias

Due to the graded distribution of Purkinje fibres, the ablation of superior conduction bundles may have a greater effect on inferior fibres. It is crucial to carefully select the ablation energy when targeting bundle branch trunks and larger branches. Additionally, the layered distribution of Purkinje fibres can lead to compound potentials during electrophysiological mapping. Different layers of Purkinje fibres in the region form these potentials. Therefore, it is important to distinguish potentials recorded during ablation from those recorded during mapping and to select the appropriate ablation energy based on the target layer. The amplitude of the Purkinje fibres' potential is related to their cross‐sectional area. The left ventricle has a greater distribution of myocardium, resulting in the Purkinje fibres on the endocardial surface of the left ventricle being thicker than those in the right ventricle, making them easier to record. This may also be one reason why the endocardial Purkinje potentials of the left ventricle are more readily recorded than those of the right ventricle. Furthermore, the networked distribution of Purkinje fibres allows impulses to bypass other side branches of the conduction network and excite their inferior regions, even when the conduction bundle is disrupted. During ablation, it is essential to consider the extent of ablation to minimize the impact on myocardial excitation.

The networked distribution of conduction bundles allows the formation of loops of varying sizes in both directions. When Purkinje fibres are damaged by factors such as ischemia, hypoxia, hyperglycemia, or mechanical stretch, certain regions experience a decrease in conduction velocity, leading to the formation of slow conduction zones. These zones can form re‐entry circuits with the intact Purkinje fibre network, potentially contributing to the development and maintenance of ventricular arrhythmias.^6^, ^13^, ^14^ Once an arrhythmia dependent on this mechanism has been identified by electrophysiological mapping, a strategy called ‘de‐networked’ Purkinje ablation can be applied. This approach involves dividing the Purkinje fibres on the ventricular endocardial surface into distinct regions, reducing the likelihood of re‐entry between Purkinje fibres while minimizing the impact on effective myocardial excitation in that area. Previous studies have shown the effectiveness of ‘de‐networking’ the Purkinje fibre network on the endomyocardial surface in the treatment of certain ventricular arrhythmias.^3^, ^15^ In this procedure, block lines are typically arranged in a ‘T’ or ‘X’ shape. Our study of the distribution characteristics of Purkinje fibres provides an anatomical basis for the implementation of this technique.

In cases of ventricular chamber dilatation due to various physiological and pathological causes, certain ventricular arrhythmias may be related to the deformation and mechanical stress experienced by the false tendons. Studies have suggested that the region of the endocardium where the false tendons attach may be more susceptible to mechanical stretch, particularly due to the denser connective tissue sheath in the mid‐segment of the false tendons. Therefore, ablation at the junction between the false tendons and the endocardium has been suggested as a potentially effective treatment.^6^, ^13^, ^14^ With the guidance of intracardiac ultrasound, precise positioning of intracardiac structures is available, enabling the ablation of structures such as tendons.^16^ However, further investigation is required to understand the effect of mechanical stretch on the electrophysiological activity of this junction.

In recent years, LBB pacing has been increasingly utilized as a method for correcting complete LBB block and treating heart failure. LBB pacing is typically performed by placing an electrode from the right heart into the left heart after recording his bundle potentials in the interventricular septum of the right ventricular. This study elucidates that the LBB trunk and proximal branches have a broad distribution, allowing the pacing electrode to capture the left ventricular conduction system with a relatively low threshold. Adjusting the orientation of the pacing electrode during the implantation process provides significant guidance for locating the LBB and identifying the ideal pacing site.

## 5 LIMITATIONS

Overall, using multiple staining methods and both gross and microscopic observations, the study demonstrated the distributional characteristics of graded, layered, and networked distribution of canine left ventricular Purkinje fibres. These findings provide, to some extent, an anatomical basis for electrophysiological study, mapping, and catheter ablation therapy. However, it is important to acknowledge several limitations of this study. Firstly, the anatomical features of the canine endocardium can vary significantly among different breeds, regions, and individuals, and therefore, the comprehensive coverage of these features in this study may be limited. Secondly, this study only focused on describing the anatomical features and did not calibrate or observe the electrophysiological properties. Finally, the study specifically examined the left ventricular endocardium and did not further investigate the distribution of right ventricular Purkinje fibres.

The study, utilizing multiple staining methods and both gross and microscopic observations, revealed that the distribution of canine left ventricular Purkinje fibres exhibits graded, layered, and networked characteristics. This finding provides an anatomical basis for electrophysiological study, mapping, and, to some extent, catheter ablation therapy.

## AUTHOR CONTRIBUTIONS

**Yunhao Li:** Conceptualization (equal); data curation (equal); writing – original draft (lead). **Daoyang Zhang:** Conceptualization (equal); data curation (equal); methodology (equal). **Yunfan Meng:** Data curation (equal). **Jie Zhang:** Conceptualization (equal); writing – review and editing (equal). **Qi Zhang:** Data curation (equal); investigation (equal). **Ping Zhang:** Writing – review and editing (equal). **Yujie Zhang:** Methodology (equal); software (equal). **Mingyu Sun:** Writing – review and editing (equal). **Zulu Wang:** Conceptualization (equal); supervision (equal). **Ming Liang:** Conceptualization (lead); supervision (lead); writing – review and editing (lead).

## FUNDING INFORMATION

This research did not receive any specific grant from funding agencies in the public, commercial, or not‐for‐profit sectors.

## CONFLICT OF INTEREST STATEMENT

The authors declare that they have no known competing financial interests or personal relationships that could have appeared to influence the work reported in this paper.

## Data Availability

Data are available within the article or its supplementary materials. The authors confirm that the data supporting the findings of this study are available within the article or its supplementary materials.
