# Correction to “Distribution characteristics of Purkinje fibres in the canine left ventricle”

**DOI:** 10.1111/jcmm.70164

**Published:** 2024-11-12

Li Y, Zhang D, Meng Y, et al. Distribution characteristics of Purkinje fibres in the canine left ventricle. J Cell Mol Med. 2024;28(18):e70117. doi: https://doi.org/10.1111/jcmm.70117.

In Figure 1A and 1B, the positions of ‘LAB’ and ‘LPB’ in the images should be swapped. The modified figure is as follow.

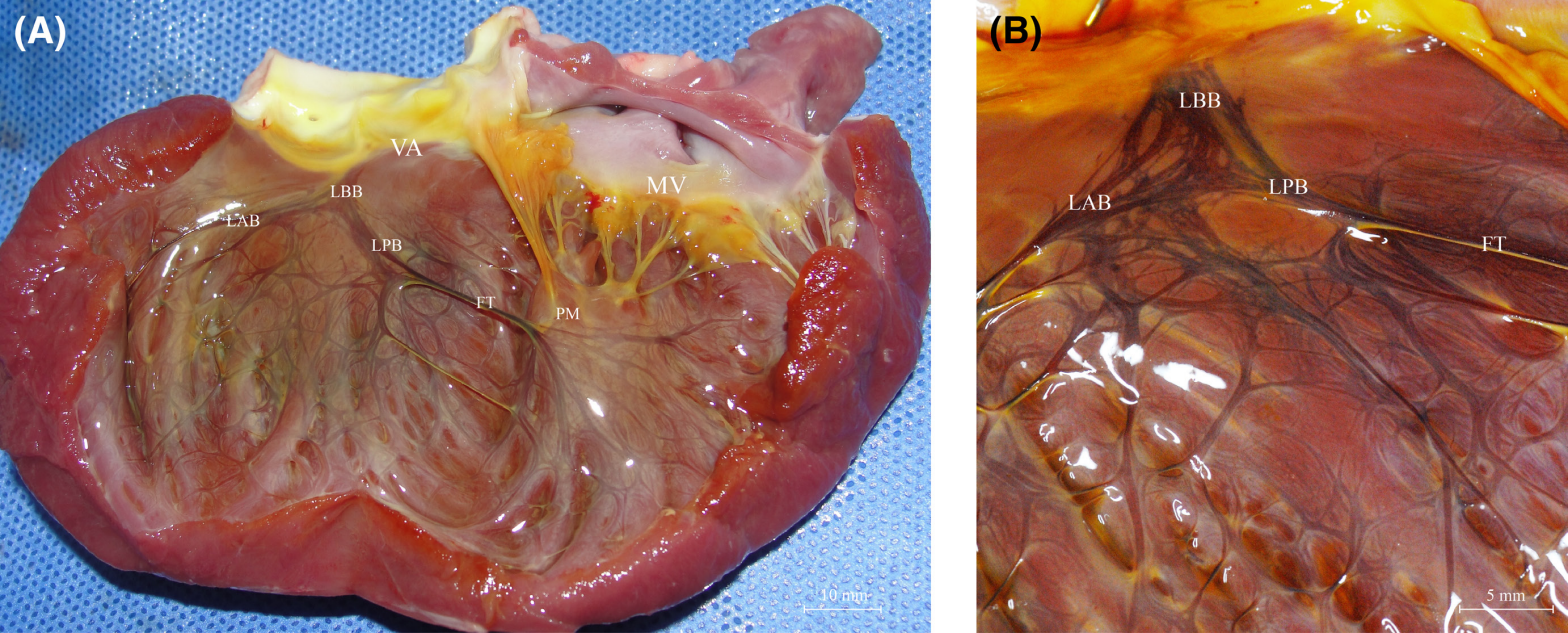

We apologize for this error.

